# Giant urinary bladder stone: an uncommon cause of anuria

**DOI:** 10.11604/pamj.2024.48.47.42469

**Published:** 2024-06-07

**Authors:** Hamedoun Larbi, Alami Mohammed

**Affiliations:** 1 Service of Urology, Military Hospital of Instruction Mohamed V, Hay Ryad-10100, Rabat, Morocco

**Keywords:** Renal failure, anuria, giant urinary bladder stone

## Image in medicine

The patient was 73-year-old, with antecedents of diabetes, arterial hypertension, atrial fibrillation on anticoagulation, and benign prostatic hypertrophy for more than 10 years on medical treatment with therapeutic malobservance. He was admitted to the intensive care unit for management of an acute subdural hematoma. On admission, the patient was comatose, had a Glasgow score of 8, intubated and ventilated, and the brain scan already indicated the beginnings of brain engagement. He underwent drainage of the hematoma after being placed in the appropriate condition. Forty-eight hours later, the patient presented with anuria, and the work-up showed an acute renal failure with creatinine at 134 mg/l, urea at 2.98 g/l, alkaline reserve at 9 mmol/l, hyperkalemia at 7 mmol/l and a GFR of 4. This prompted the intensive care doctor to order an emergency abdominal scan, which revealed significant bilateral pyelocalic dilatation with the presence of 2 huge bladder stones measuring 7 and 6 cm each. Given the anuria and fragile condition, the patient was dialyzed and then admitted to the operating room for drainage using a bilateral double J catheter, which was very difficult given the macromolecules occupying almost the entire bladder. Postoperatively, the patient resumed diuresis and began to improve his renal function, but unfortunately, his prognosis was poor following worsening cerebral involvement and he died within 72 hours. The association of a giant primary calculus with acute renal failure is very rare, which is what makes our observation so special.

**Figure 1:**
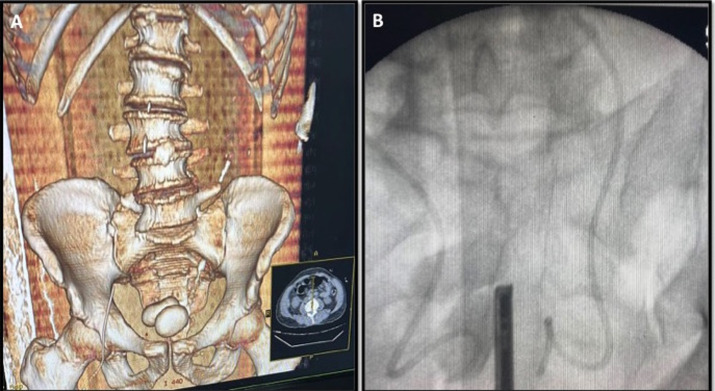
A) abdominal and pelvic CT scan showing two huge bladder stones; B) two huge bladder stones after the JJ probes have been placed using an image intensifier

